# Willingness to Participate in Clinical Trials among Patients of Chinese Heritage: A Meta-Synthesis

**DOI:** 10.1371/journal.pone.0051328

**Published:** 2013-01-17

**Authors:** Alexander Limkakeng, Amruta Phadtare, Jatin Shah, Meenakshi Vaghasia, Ding Ying Wei, Anand Shah, Ricardo Pietrobon

**Affiliations:** 1 Division of Emergency Medicine, Department of Surgery, Duke University, Durham, North Carolina, United States of America; 2 Department of Surgery, Duke University, Durham, North Carolina, United States of America; 3 Department of Radiation Oncology, University of Pennsylvania, Philadelphia, Pennsylvania, United States of America; 4 National Cancer Centre, Singapore, Singapore; 5 Department of Surgery, Duke University, Durham, North Carolina, United States of America; University of Ottawa, Canada

## Abstract

**Background:**

Subjects of Chinese heritage have been found to participate in clinical research at lower rates than other groups despite growing in numbers as a population. While much research has examined research participants' motivation, there has not been a comprehensive synthesis of this information with respect to participants of Chinese descent. We sought to identify the factors that promote and hinder participation in clinical research among participants of Chinese heritage.

**Methodology/Principal Findings:**

We conducted a systematic review of the literature in Pubmed, OpenJGATE, SCIRUS, and COCHRANE databases and performed a meta-synthesis of retrieved articles. We extracted qualitative data, such as quotes to identify emerging themes. We identified five studies that met our selection criteria. Of them, only one (1/5) was conducted in China while other studies involved Chinese emigrants in USA (3/5) and Singapore (1/5). Participants from China were similar to emigrants with regard to factors that either promoted or decreased research participation. Four studies reported data exclusively on Chinese subjects. Three of the five studies involved qualitative interviews while the others were conducted using a survey design. Six themes favoring research participation were identified: Personal Benefit to Participants, Financial Incentives, Participant Sense of Altruism, Family or Physician Recommendations, Advertisements, and Convenience to the Participant. Five factors were seen as a barrier to participation in clinical trials: Mistrust of Researchers, Language Barrier, Lack of Financial and Other Support, Cultural and Social Barriers, Lack of Knowledge about Clinical Trials.

**Conclusions/Significance:**

Chinese heritage clinical research participants value personal benefit, financial incentives, the ability to help others, recommendations of others, advertisements, and convenience when considering clinical research participation. In addition, the establishment of trust and addressing knowledge deficits are important factors to them. Investigators seeking to optimize enrolment in these populations should incorporate these findings into their study design and subject handouts.

## Introduction

The importance of subjects of Chinese heritage is increasing, both inside and outside of China. Subjects living in China are being increasingly sampled due to the globalization of clinical research [1], [2]. In a sample of 150 prominent clinical trials, nearly a quarter enrolled patients outside the United States and Europe, [1] which is a major shift. China's average relative annual growth rate for research (47%) is the highest among the 25 nations who are active in research [2]. Second, subjects of Chinese heritage are important *outside of* China because the National Institutes of Health and other sponsoring agencies require researchers to recruit subject samples reflective of their communities at large [3]. In the US alone, China has consistently been one of the top sources of immigrants for the last decade, with a Chinese-American population numbering approximately 3.8 million [4]. Asian-Americans have been underrepresented in US research [5], [6], [7].

Examining the factors that affect Chinese heritage patients' willingness to participate in clinical research is therefore important for medical, ethical, and financial reasons [8]. Findings in non-Chinese heritage populations may not apply to Chinese heritage patients. As an example, in a large cohort of 3 major Asian ethnicities, the original GRACE risk stratification score, (derived from populations outside of Asia), was found to underestimate in-hospital mortality after myocardial infarction [9]. The Belmont Report [10] calls for justice as a guiding principle in subject recruitment, which means that subjects of certain ethnic backgrounds should not be recruited differentially. Thus, understanding subjects' concerns about participation would increase ethical recruitment of individuals of Chinese descent. And finally, many clinical trials are delayed due to lack of enrolment. So understanding Chinese heritage subjects' concerns about participation would increase the efficiency of recruitment.

The clinical trial process and factors affecting participation has been well described in Western nations [11]–[23]. Prior work has also examined factors associated with research participation in emerging-economy cultures such as Brazil and India. Such trials found that personal health benefits offered by the trial and a sense of altruism contributed to research participation, whereas there were a variety of barriers including mistrust of researchers, fear of side effects of the intervention, and inconvenience to the subject [24]–[28]. However, it is well accepted that motivations for research participation vary between patients of different cultural backgrounds [29]–[32]. Therefore, we conducted a systematic review and synthesis of qualitative research studies that report the motivations for and concerns about participating in clinical research of subjects of Chinese heritage.

## Methods

### Research question

We carried out a systematic review of literature published between 1985–2009 to understand Chinese patients' motivations and concerns to participate in clinical trials.

### Ethics

We did not apply for ethics approval as we conducted a systematic review and meta synthesis based on published literature.

### Search Strategy

Three independent reviewers (AP, MV, YW) carried out a systematic search in the following online free databases – Pubmed (1985–2009) [33], OpenJGATE (1985–2009) [34], SCIRUS (1985–2009) [35]. We also carried out the same review in COCHRANE (1985–2009) [36] which is a paid database. We made use of the following keywords independently or as a combination while searching these databases: Patient participation, Chinese, China, subject participation, concerns, attitudes and participation and Chinese, Asian, willingness to participate. A fourth reviewer (JS) unaware of the research question carried out a blinded review in the same databases. All reviewers (except YW) have previous experience with conducting systematic reviews. We included a reviewer fluent in Chinese language (YW) expecting to find articles in Chinese language. Based on our results, we created a list of Medical Subject Headings (MeSH) Terms and used them separately or in combination to search the databases listed earlier. We used the “related article” tool in Pubmed to identify articles similar to a given article matching our quest. [37] Next we reviewed the bibliography of all relevant articles identified during our initial search. In order to keep ourselves updated with any new articles within the time we published this article, we created Really Simple Syndicate (RSS) feeds for our search strategies. Details of the search strategy are available in Supporting Information – S1.

### Selection criteria

#### Inclusion criteria

We framed selection criteria to filter through the literature search results and shortlist articles that would help us answer our research question. These criteria included:

Studies involving patients/subjects (in contrast with subjects that were not being recruited for real trials); Studies of Chinese nationals or an individual of Chinese origin; studies using experimental (trials) or qualitative methods (interviews, focus groups, ethnographic, or survey) to collect data; studies whose outcome measures included factors affecting participation in clinical trials. For the purpose of this article, we refer to subjects who were born in China as subjects of Chinese heritage (heritage meaning “the intangible attributes of a group or society”). People sharing a common heritage have common beliefs and therefore should have similar responses when requested to participate in clinical trials. We included trials conducted both within and outside of China and analyzed them separately for comparison. Finally, we chose to focus on full text articles only.

### Exclusion Criteria

We excluded studies that met the following criteria: 1. Studies that did not directly evaluate subjects/patients but rather, evaluated factors influencing their participation by analyzing retrospective clinical trial data, 2. studies that evaluated other populations, unpublished articles, dissertations, and abstracts without full text.

As a next step, we reviewed and screened through articles retrieved during the initial literature search exercise. The articles were screened by title, abstract and full text. At each step, we excluded articles that did meet our criteria.

### Communication with authors

We sent emails to the authors of shortlisted articles explaining our study and inquiring about the existence of other literature that could help us answer our research question.

### Data abstraction

Two of us (AP, MV) independently carried out data abstraction from the shortlisted articles and populated it in a spreadsheet. We extracted information about factors favoring and factors serving as barriers to participation in clinical trials in 2 separate sheets. Both the reviewers shared their spreadsheets and resolved discrepancies by mutual consensus.

### Quality Analysis

Before carrying out a meta-synthesis of qualitative studies retrieved during our review, we used the RATS Scale [38] to evaluate the quality of the shortlisted articles. One of us (AP) with previous experience with RATS scale evaluated each shortlisted article and assigned scores on a LIKERT scale [39]. The scores ranged between 1 to 3, where 1 meant ‘highly approved’ and 3 meant ‘least approved’. The scores were then evaluated by a statistician.

### Study characteristics

After reviewing the shortlisted articles in detail, we extracted descriptive data to help us summarize them. We captured details about age, ethnicity, location of study, details of outcomes and intervention (including study questionnaires) in a spreadsheet.

### Qualitative data synthesis

We observed that shortlisted articles either reported participant quotes from the qualitative interviews that they conducted or reported the percent results for each question from the survey that they undertook. We extracted each of these quotes and percent responses and populated them in a spreadsheet. Two reviewers (AP and MV) reviewed the spreadsheet independently and categorized the results, eventually attempting to identify emerging themes. Disagreements were resolved by discussion and mutual consensus. The final spreadsheet was reviewed by an epidemiologist (RP) to resolve any discrepancies. We identified and furnished each emerging theme with quotes from individual studies. Finally, we categorized the emerging themes into two groups: Factors favoring the participation and factors serving as barriers for participation in clinical trials. We performed a post-hoc sub analysis of studies conducted in China versus outside of China to account for cultural context in which the studies were being performed.

## Results

We identified a total of five manuscript articles that met our inclusion and exclusion criteria. Detailed analysis of each article helped us identify themes motivating/serving as barriers to Chinese individual's participation in clinical trials. (Table 1 and Table 2) We analyzed the studies conducted outside of China initially with the one conducted in China. We later separated the one study conducted in China from the others and present it separately within the same framework of the Results.

**Table 1:** Factors favoring participation in clinical trials.

Study title	Personal Benefit	Incentives	Altruism	Recommendation	Advertisement	Convenience
Clinical Trials: Understanding and Perceptions of Female Chinese-American Cancer Patients-	No other effective treatments (10%)	No data	Altruism (8%)	Recommended by trusted individual (including oncologist) (9%)	No data	Not harmful to the body (3%)
Concerns over participation in genetic research among Malay-Muslims, Chinese and Indians in Singapore - a focus group study	Quotes present	No data	No data	No data	No data	Quotes present
Susceptibility of Elderly Asian Immigrants to Persuasion With Respect to Participation in Research -	Update information about HIV/AIDS: (98.3%)Free HIV counselling and testing (98.3%)Motivation to avoid risky behaviours(98%)Protection against HIV infection: (99%)	If they receive money in return (19%)	No data	If landlord request (15%)If their physician ask them to (25%)	If they see advertisement in newspaper (11%)	No data
Understanding Immigrant Chinese American participation in Cancer screening and Clinical trials	if they are sick they will participate	No data	No data	On recommendation of doctors	No data	geography/distance, education, time
Willingness of Chinese injection drug users to participate in HIV vaccine trials	Update information about HIV/AIDS Free HIV counselling and testing (0.43) Protection against HIV infection	Incentive for participation: (99.3%)	Wish scientists develop an effective vaccine	No data	No data	No data

**Table 2:** Factors serving as barrier to participation in clinical trials.

Study title	Mistrust	Language Barrier	Lack of Support	Cultural and Social Barriers	Lack of knowledge
Clinical Trials: Understanding and Perceptions of Female Chinese-American Cancer Patients-	Quotes present	Language Barrier (80%)	Lack of financial and other support (2%)	Quotes present	Lack of knowledge (24%)Negative Attitudes Insufficient information (3%)More information (6%)Quotes present
Concerns over participation in genetic research among Malay-Muslims, Chinese and Indians in Singapore - a focus group study	Quotes present	No data	No data	No data	No data
Susceptibility of Elderly Asian Immigrants to Persuasion With Respect to Participation in Research -	No data	No data	No data	No data	No data
Understanding Immigrant Chinese Americane' participation in Cancer screening and Clinical trials	Quotes present	Quotes present	No data	Other cultural considerations.	Lack of knowledge of trials
Willingness of Chinese injection drug users to participate in HIV vaccine trials	Vaccine probably does not work (73.5%)Vaccine may cause health problems (55.4%)Vaccine may cause health problems: (74.8%)Develop a false positive HIV test (74.8%)Weaken the body's ability to fight off HIV (53.7%)	No data	Family would support me to participate in HIV vaccine trials (76.2%)	People may think I'm at high risk for HIV (40.6%)People may think I'm infected with HIV (37.9%)People may refuse to have sex with me (37.6%)People may avoid social contact with me (37.6%)	No data

**Note:** “Quotes present” indicates that there exist quotes in these studies which support the emerging themes.

### Search Strategy Results

**Figure 1:**
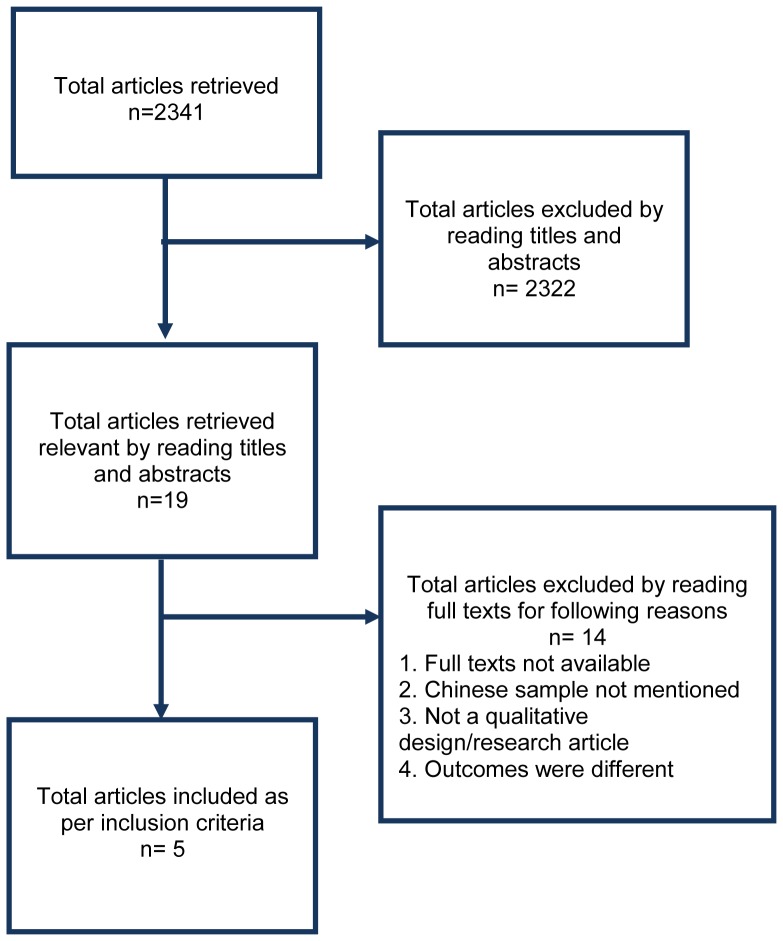
Study plan.

Our initial review of the literature yielded 2341 articles. After reviewing titles and abstracts from the 2341 articles, we shortlisted 19 articles that were relevant to our research question. We excluded 14 articles due to one of the following reasons: 1. Unavailability of full text, 2. Chinese sample not mentioned, 3. The article did not have a qualitative research design and 4. Outcomes assessed differed when compared with our research question. (Figure 1)

### Quality Analysis

Analysis of LIKERT scale scores indicated that all five shortlisted articles received scores between 1–2. As a consequence, we included all five articles in our meta synthesis.

### Study characteristics

Of the five studies [7], [40]–[43] included in our Meta synthesis, three were conducted in USA while the remaining were conducted in China and Singapore respectively. One study compared Chinese-American immigrants to non-Chinese participants, the rest focused exclusively on Chinese heritage subjects. Three of the five studies involved qualitative interviews while the others were conducted using a survey design. All of them were conducted among adult individuals with two studies evaluating the elderly [41], [43].

### Validity assessment

The list of articles independently retrieved by the fourth third blinded reviewer (JS) matched those retrieved by the primary reviewers (AP, MV, and YW) thus validating the review results.

### Emerging themes: Factors serving as motivation for participation in clinical trials

#### Personal Benefit

We observed that personal benefit was the primary driver influencing the participation of Chinese individuals in clinical trials. Participants from four of the five trials [7], [41]–[43] reported this theme. This was especially true for those who were already sick and did not have access to any other effective treatment. It is apparent from the quote “if they are sick they will participate” [41]

In the one trial conducted in China, willingness was also stronger when individuals were convinced about the effectiveness of treatment being provided within the clinical trial.

In the HIV patient populace, willingness to participate was nearly 74.3% [42]. Access to updated and latest information about HIV/AIDS treatment, free HIV counseling and testing and lack of effectiveness in existing treatment methods were some factors responsible for higher inclination towards participation in clinical research. Healthy Chinese subjects were equally inclined to participate due to the protection offered against HIV infection and motivation to avoid risky behavior.

Similarly, potential participants in a genetic research study were interested to know whether the study team would share details of their diagnosis and provide free treatment. This can be seen from the excerpt: “What happens if you find something wrong with me? Will you let me know? Will you give me free treatment?” [40]

### Incentives

One of the four studies conducted outside of China and the only trial conducted in China [42], [43] reported the influence of incentives on their willingness to participate in clinical trials. Preference for incentives was reported by nearly all participants from one of these studies. 99.3% [42], Participants from the second study reported their preference for money as an incentive to participate in clinical trials [43].

### Altruism

Participants from one of the four studies conducted outside of China and the only trial conducted in China [7], [42] reported altruism as the factor influencing their decision to participate in clinical trials. Notably, in the study conducted in China, all participants confirmed their intention to participate in clinical trials so that scientists can prepare better and more effective vaccines [42].

### Recommendation

Recommendation from trusted individuals like relatives, friends, and family physician/doctors was a dominant theme across three of the five studies [7], [41], [43]. Influence of a doctor's recommendation was quite common as can be seen from the excerpt:“If he [the oncologist] said it's needed then I will [do it].… There must be reasons for them wanting me to participate. … If the doctor asked me to go [for the treatment being researched], I would go.” [7] Some individuals even felt that the guidance and motivation to participate should originate from the treating physician. This is apparent from the excerpt: “Yes I would [participate in clinical trials]. [7] Actually it really should be up to the doctor. If the doctor gives you confidence, I think you would participate.” Others preferred to follow their doctor's advice as they did not understand the details and trusted their doctor to guide them. For example: “I will listen to the doctor, because I don't understand – really I don't understand.” and “I will listen to the doctor. If he wants me to try the new [medicine], I will try [it].” [7] In some cases (15%) a recommendation from the landlord was also given importance [43].

### Advertisement

Advertisement in newspapers was also noted as an important factor in one of the five studies (11%) [43].

### Convenience

Personal convenience while participating in clinical research was afforded importance by participants. They preferred research studies that were 1. located nearby, 2. studying interventions that were not harmful to the body and did not require significant amount of education/training. [7], [41]. For example one participant preferred to participate in the absence of any injury to his person. “As long as there is nothing hurting you, then it's okay. You can try it… If it does not cause a big harm to the body, then it's okay.” [7] Others preferred to participate if it did not require a significant amount of time investment as is apparent from “Is my participation just one day off event or the team will come and disturb me again for follow up” (Chinese female secretary). [40]

Similarly, they also had concerns about the location of blood collection and its volume. For example, participants preferred to avoid blood collection at home, citing security concerns. “Where will you be taking our blood specimens? I prefer that your staff do not come to our home to take our blood, who knows he may be a conman and inject some drugs in the my body as well as make me drowsy. I may be robbed” (Chinese female assistant nurse)” [40] Others were worried that a significant volume of blood would be withdrawn from their body: “Will you be taking whole syringe of blood? That is a lot….. ”(Chinese female food vendor) [40].

### Emerging themes: Concerns about participation in clinical trials

#### Mistrust

Mistrust was a prominent barrier to trial participation among Chinese individuals. Four of the five studies, including the study conducted in China, [7], [40]–[42] highlighted its role. Lack of knowledge and understanding of clinical trials led to mistrust and non participation. It was conveyed through excerpts like “I think it would be very difficult [for Chinese people to agree to participate in clinical trials because] ….they don't know what you are doing” [7]. Mistrust and suspicion was more prominent in Chinese immigrants who did not understand what was involved and hence were difficult to persuade. This is apparent from the excerpt: “Like those [who] just came from China, those who just immigrated, they may not want to [participate in clinical trials]. Because they still don't know what you are going to do.… They are not easily persuaded.… Why they are like this? Is this doctor capable or not? Suspicious!… Don't know whether or not the translation is enough.” [7] Others communicated concern about the safety of their personal information and whether they could seek legal recourse in such a situation through excerpts like “What happens if the information about my disease leaks out ? Can I sue the research body or government?” [40] Others were paranoid in working with strangers and safety of their personal information. For example, “I don't see any reason why I should give my blood to complete strangers. I don't even know you. How do I know what you will do with my results? What if the information leaks out to my employer or insurance company?” [40]

In HIV vaccine trials, a majority of the participants were not sure whether the intervention would actually work. Others also believed that the HIV vaccine may cause health problems or cause one to develop a false positive HIV test. Some perceived that the HIV vaccine would reduce their immunity, thus rendering them susceptible [42].

### Language Barrier

Language barrier was highlighted in two out of five studies [7], [41]. Notably, most of the participants in one of the two studies believed that language of communication significantly influenced their decision to participate in trials. Among immigrants, lack of formal education was identified as a major factor influencing participation in trials. It is apparent from excerpts like “Many new immigrants from Fuzhou don't have a formal education, never learned Mandarin…only 3% are elementary educates, 97% are non-educated.” [41] Others reported that translation of consent and protocol documents was of little use as many potential participants were either uneducated or did not understand the technical language used: “…they don't know how to communicate with the investigator…even if we have [consent and protocols] translated in Chinese…they don't know how to read or understand the [technical] language.” [41]

### Lack of Support

Lack of financial and other support (2%) [7] was also reported as a barrier to participation. In the HIV vaccine trial conducted in China, respondents were willing to participate if their family extended support. (76.2%) [42].

### Cultural and Social Barriers

Chinese individuals afforded significant importance to social and cultural factors. Accordingly, they had second thoughts about participation in clinical trials if their cultural beliefs were not satisfied: “they won't pay attention [to].… It's more difficult, especially with Chinese people, because we don't have this custom [to] volunteer!” [7] This was more apparent in the HIV vaccine trial conducted in China. Being at high risk for HIV or being infected with HIV would result in lower social and physical contact with others. [42]

### Lack of knowledge

Limited previous information or details about clinical trials was seen as an important barrier to participation. In many cases this led to an overall negative attitude to clinical research participation. In other instances, participants requested more information before taking a decision. Many potential participants lacked any knowledge of clinical research (62%). [7], [41] In some cases individuals equated all research with qualitative interviews. For example: “Seems like [they] interview some individuals to see [what] their experience [is], just like you interview me right now. I think it's like that, isn't it?” [7] In other cases, potential participants believed that clinical trials were conducted when the prognosis was poor and doctors preferred not to pursue treatment. For example: “So, my feeling is that, when doctors mention a clinical trial, it seems like [they are] going to give up on you.” [7]

### Sub-group analysis summary

Only one of the studies included in the meta synthesis was conducted in China [42]. It reported Personal benefit, Incentives, and Altruism as factors motivating participation and Mistrust, Lack of support, and Cultural & social barriers as barriers to participation in clinical research. Other studies were conducted with participants who were of Chinese origin but not living in China at the time of the study.

## Discussion

We have conducted the first systematic review of factors affecting willingness to participate in clinical trials among subjects of Chinese descent. The perception of personal benefit and convenience, a sense of altruism, advertisements, and favorable recommendations from physicians and family increased the likelihood of Chinese heritage subjects participating in clinical research. On the other hand, concerns negatively impacting participation included lack of complete information, language barriers, lack of social or financial support, cultural values, and mistrust of research.

Many of the themes in this study have appeared in prior studies of research participation, although the relative importance of themes varied. For example, studies in a variety of Western settings demonstrate the importance of altruism [24], [25], [44]. Our group's study of Indian subjects found that a sense of altruism was a recurring theme as well [29]. This is especially interesting when it is recognized that compensation is also a frequently acknowledged motivator [45]. Thus, it seems that even if a subject is compensated, the sense that one is helping society is still an important benefit of participating in research. A second common motivator was convenience to the subject. In light of subjects' perception that they are undertaking an altruistic endeavor, it follows that they do not feel that they should be inconvenienced. Although it was worded in slightly different terms, many studies have demonstrated the phenomena that subjects considered the relative convenience (or lack thereof) of their participation [24], [25], [44].

On the other hand, mistrust of researchers was a near-universal barrier to research participation across cultures. Multiple studies in the United States have identified racial disparities in research participation [46], and in large part this has been attributed to a lack of trust in medical researchers [29], [32]. Some of this mistrust may be the legacy of past misconduct (e.g., Tuskegee) by researchers or by the medical community at large. Conversely, for those who trusted their physician, a great deal of weight was placed on the physician's recommendation, with some subjects indicating a willingness to completely abdicate decision making to their physician. This willingness was related to subjects' perception that they did not have the knowledge needed to make a decision. Although this has also been cited as a reason for participation in other cultures [47], in one of the studies included in our meta synthesis [43], this finding was statistically more likely among Chinese heritage participants.

These findings demonstrate shared values between cultures with regards to research (Table 3). They also point the way to clinical research recruitment success for researchers. Investigators should prioritize the incorporation of benefits (personal and financial), convenience of the trial to participants, and communicate the trial's benefit to society. In addition, the establishment of trust and addressing knowledge deficits are important factors.

**Table 3:** Characteristics of articles included.

Study title	Country	Subject Population considered	Time since immigration, if relevant, mean in years	Language of interviews	Intervention	Age group	Factors evaluated
Clinical Trials: Understanding and Perceptions of Female Chinese-American Cancer Patients-	USA	34 Chinese American individuals	20.8	Chinese (95% of participants)	Semi structured interview	20–85 yrs	Understanding and perceptions of clinical trials barriers and facilitators to clinical trial participation
Concerns over participation in genetic research among Malay-Muslims, Chinese and Indians in Singapore - a focus group study	Singapore	35 Chinese individuals	Not Available	Not Available	Focus Group Discussions	18 Years and above	Concerns over participation in genetic research
Susceptibility of Elderly Asian Immigrants to Persuasion With Respect to Participation in Research	Boston, USA	immigrant Chinese (Asian, n = 79) and the non-immigrant non-Chinese (Non-Asian, n = 58)	14	Cantonese	Written surve	Elderly	Influence from their children, landlords, physicians, a newspaper ad, and an offer of money with regard to participation in a research study.
Understanding Immigrant Chinese Americans' participation in Cancer screening and Clinical trials	Manhattan Chinatown community, USA	11 physicians, 15 community leaders, and 38 community members	Less than 10 years: 35% >10 years: 65%.	Not Available	Focus Group Discussions	<50 and 50 and above	First generation Chinese American attitudes and behaviors toward cancer, cancer screening and prevention, clinical trials and barriers to participation in cancer screening clinical trials
Willingness of Chinese injection drug users to participate in HIV vaccine trials	Northwestern China	401 Injection Drug Users	Not Available	Chinese	Cross-sectional-Questionnaire interviews	18 years or older	Willingness to participate (WTP) in HIV vaccine trials among Chinese IDUs

This is a systematic review of existing literature. It does not include factors that have not been identified in the peer-reviewed literature. Although we have done an extensive systematic search, it is possible that relevant studies may have been excluded. By their very nature, qualitative studies have limited sample sizes. Thus, it is possible that the existing literature may reflect an unrepresentative sample of Chinese heritage subjects. In addition, the studies included in this review use interviews to obtain data. It is possible that the real factors motivating research participation are not what subjects say that they are. Subjects might be unwilling to reveal their actual motivations or their relative importance for fear of stigmatization or shame (for example, the relative importance of compensation). Alternative, experimental approaches would be required to confirm the importance of these factors.

During our review, we retrieved four studies involving subjects of Chinese origin who did not live in China at the time of the research. Only one study involved participants living in China. Despite this difference, the themes derived were similar in both cases.

Other trials have likewise considered immigrants to have similar values as Chinese subjects actually located in China [48]. Indeed, one of the studies in our meta synthesis noted marked statistically significant differences in attitudes between immigrant Chinese and non-Asian elderly [43]. Many of the participants from the studies included in our meta synthesis were older adults, whose values and opinions toward research were likely well established prior to their immigration and less influenced by their geographic setting. Furthermore, upon sub analysis, location of the study did not appear to greatly influence results, suggesting a commonality of values between Chinese residents and emigrants.

## Conclusions

In closing, we have identified Chinese heritage subjects' motivations for and concerns about clinical trial participation. The similarities between the present study and previous evidence suggest a commonality among diverse cultures and, possibly, universality. This information can be used to interpret existing data and plan future trials in Chinese populations. There is a dearth of literature exploring how altering various factors in an experimental fashion would affect enrolment in these cultures. Additionally, although the factors listed are what participants report, it is possible that there is discordance between what participants report are important and what actually affects their willingness to participate. Future studies should explore these possibilities.

## Supporting Information

Supporting Information S1 **Search strategy details for the Systematic review.**
(DOCX)
