# Supplementary material for: Willingness to Participate in Clinical Trials among Patients of Chinese Heritage: A Meta-Synthesis
Source: PLoS One. 2013 Jan 17;8(1):e51328. doi: 10.1371/journal.pone.0051328 (PMC3547937; doi:10.1371/journal.pone.0051328)
Supplement: Supporting Information S1 — Search strategy details for the Systematic review. (DOCX) [file pone.0051328.s001.docx]

**Supporting Information**

**S1 - Search strategy**

(("patient participation"[MeSH Terms] OR ("patient"[All Fields] AND "participation"[All Fields]) OR "patient participation"[All Fields]) AND ("asian continental ancestry group"[MeSH Terms] OR ("asian"[All Fields] AND "continental"[All Fields] AND "ancestry"[All Fields] AND "group"[All Fields]) OR "asian continental ancestry group"[All Fields] OR "chinese"[All Fields])) AND ("loattrfull text"[sb] AND "loattrfree full text"[sb] AND "humans"[MeSH Terms] AND English[lang])

(("patient participation"[MeSH Terms] OR ("patient"[All Fields] AND "participation"[All Fields]) OR "patient participation"[All Fields]) AND ("china"[MeSH Terms] OR "china"[All Fields])) AND ("loattrfull text"[sb] AND "loattrfree full text"[sb] AND "humans"[MeSH Terms] AND English[lang])

("1985"[PDAT] : "2009"[PDAT]) AND (subject[All Fields] AND participation[All Fields] AND ("asian continental ancestry group"[MeSH Terms] OR ("asian"[All Fields] AND "continental"[All Fields] AND "ancestry"[All Fields] AND "group"[All Fields]) OR "asian continental ancestry group"[All Fields] OR "chinese"[All Fields])) AND ("humans"[MeSH Terms] AND English[lang])

(concerns[All Fields] AND participation[All Fields] AND ("asian continental ancestry group"[MeSH Terms] OR ("asian"[All Fields] AND "continental"[All Fields] AND "ancestry"[All Fields] AND "group"[All Fields]) OR "asian continental ancestry group"[All Fields] OR "chinese"[All Fields])) AND ("humans"[MeSH Terms] AND English[lang])

("1985"[Publication Date] : "2009"[Publication Date]) AND (attitudes and participation and chinese)

("1985"[PDAT] : "2009"[PDAT]) AND (subject[All Fields] AND participation[All Fields] AND ("asian continental ancestry group"[MeSH Terms] OR ("asian"[All Fields] AND "continental"[All Fields] AND "ancestry"[All Fields] AND "group"[All Fields]) OR "asian continental ancestry group"[All Fields] OR "asian"[All Fields])) AND ("humans"[MeSH Terms] AND English[lang])

("1985"[PDAT] : "2009"[PDAT]) AND (subject[All Fields] AND participation[All Fields] AND ("asia"[MeSH Terms] OR "asia"[All Fields])) AND ("humans"[MeSH Terms] AND English[lang])

(willingness[All Fields] AND participate[All Fields] AND ("asian continental ancestry group"[MeSH Terms] OR ("asian"[All Fields] AND "continental"[All Fields] AND "ancestry"[All Fields] AND "group"[All Fields]) OR "asian continental ancestry group"[All Fields] OR "chinese"[All Fields])) AND ("humans"[MeSH Terms] AND English[lang])

willingness[All Fields] AND participate[All Fields] AND ("asian continental ancestry group"[MeSH Terms] OR ("asian"[All Fields] AND "continental"[All Fields] AND "ancestry"[All Fields] AND "group"[All Fields]) OR "asian continental ancestry group"[All Fields] OR "asian"[All Fields])

("1985"[PDAT] : "2009"[PDAT]) AND (concerns[All Fields] AND participation[All Fields] AND ("asian continental ancestry group"[MeSH Terms] OR ("asian"[All Fields] AND "continental"[All Fields] AND "ancestry"[All Fields] AND "group"[All Fields]) OR "asian continental ancestry group"[All Fields] OR "asian"[All Fields])) AND ("humans"[MeSH Terms] AND English[lang])

("1985"[PDAT] : "2009"[PDAT]) AND (("attitude"[MeSH Terms] OR "attitude"[All Fields] OR "attitudes"[All Fields]) AND participation[All Fields] AND ("asian continental ancestry group"[MeSH Terms] OR ("asian"[All Fields] AND "continental"[All Fields] AND "ancestry"[All Fields] AND "group"[All Fields]) OR "asian continental ancestry group"[All Fields] OR "asian"[All Fields])) AND ("humans"[MeSH Terms] AND English[lang])

("1985"[PDAT] : "2009"[PDAT]) AND (("motivation"[MeSH Terms] OR "motivation"[All Fields]) AND ("patient participation"[MeSH Terms] OR ("patient"[All Fields] AND "participation"[All Fields]) OR "patient participation"[All Fields]) AND ("clinical trial"[Publication Type] OR "clinical trials as topic"[MeSH Terms] OR "clinical trials"[All Fields]) AND ("asia"[MeSH Terms] OR "asia"[All Fields])) AND ("humans"[MeSH Terms] AND English[lang])

("1985"[PDAT] : "2009"[PDAT]) AND (("asian continental ancestry group"[MeSH Terms] OR ("asian"[All Fields] AND "continental"[All Fields] AND "ancestry"[All Fields] AND "group"[All Fields]) OR "asian continental ancestry group"[All Fields] OR "chinese"[All Fields]) AND participation[All Fields] AND ("clinical trial"[Publication Type] OR "clinical trials as topic"[MeSH Terms] OR "clinical trials"[All Fields])) AND ("humans"[MeSH Terms] AND English[lang])

("1985"[PDAT] : "2009"[PDAT]) AND (factors[All Fields] AND participation[All Fields] AND ("asian continental ancestry group"[MeSH Terms] OR ("asian"[All Fields] AND "continental"[All Fields] AND "ancestry"[All Fields] AND "group"[All Fields]) OR "asian continental ancestry group"[All Fields] OR "chinese"[All Fields])) AND ("humans"[MeSH Terms] AND English[lang])

("1985"[PDAT] : "2009"[PDAT]) AND (("asian continental ancestry group"[MeSH Terms] OR ("asian"[All Fields] AND "continental"[All Fields] AND "ancestry"[All Fields] AND "group"[All Fields]) OR "asian continental ancestry group"[All Fields] OR "chinese"[All Fields]) AND participation[All Fields]) AND ("loattrfull text"[sb] AND "loattrfree full text"[sb] AND "humans"[MeSH Terms] AND English[lang])
